# Online Perceptions of Mothers About Breastfeeding and Introducing Formula: Qualitative Study

**DOI:** 10.2196/publichealth.8197

**Published:** 2017-11-15

**Authors:** Anna Lena Wennberg, Sanna Jonsson, Josefine Zadik Janke, Åsa Hörnsten

**Affiliations:** ^1^ Department of Nursing Faculty of Medicine Umeå University Umeå Sweden

**Keywords:** breast feeding, bottle feeding, mothers, decision making, Internet, patient web portals

## Abstract

**Background:**

Although the benefits of breastfeeding are well established for babies and their mothers, many women give formula to their infants. Whether to breastfeed or to give infant formula is a complex decision to make. Many parents use the Internet to find information and support that relate to infant feeding decisions.

**Objective:**

The aim of this study was to analyze the perceptions of mothers, who are discussing the topic on Web forums, about introducing infant formula.

**Methods:**

This is a qualitative, descriptive, and cross-sectional study on online data from parenting Web forums. The text was analyzed using qualitative content analysis.

**Results:**

The analysis resulted in 1 main theme, “balancing between social expectations and confidence in your parental ability,” which is further divided into 3 themes: “striving to be a good mother,” “striving for your own well-being,” and “striving to discover your own path.”

**Conclusions:**

Breastfeeding is complex, and health care personnel can, with a more open approach toward formula, create better support for mothers by helping them to be more confident in their parental ability.

## Introduction

The benefits of breastfeeding for babies and their mothers are well established [1-3]. The World Health Organization and the United Nations Children’s Fund recommend exclusively breastfeeding for 6 months and partial breastfeeding for 2 years or beyond [4,5]. Many countries, such as Sweden, have followed these recommendations [6].

Breastfeeding rates differ globally, where low- and middle-income countries have the highest breastfeeding rates. In high-income countries, the prevalence varies a great deal between countries [2]. Formula feeding is most common in Western Europe, Australia, and North America, and its frequency is affected by the marketing and the availability of formula [7]. In Sweden, marketing of infant formula is regulated by the international code [4,8].

Breastfeeding is the most common way to feed the child in Sweden, with 96% of the women breastfeeding (exclusively or partly) at 1 week of age and 63% at 6 months [9]. The Swedish child health services promote breastfeeding and inform the parents about breastfeeding advantages and the negative impact on breastfeeding that formula may present [6].

A woman’s decision to breastfeed is a complex one [10], influenced by a convergence of factors that include sociodemographic and psychosocial aspects as well as historical and cultural factors [7]. Breastfeeding can be experienced as difficult, and although many mothers have expressed a need for support, they feel that they do not always get the support they need from health care professionals [11,12]. A support system may have a positive effect on the initiation and duration of breastfeeding [13]. Different interventions regarding breastfeeding support have shown to increase breastfeeding duration [7,14-16], including using the Internet [17,18]. Many parents use the Internet as support in their parenthood, and for many, it is the firsthand choice to find information about pregnancy and parenthood, including infant feeding [19-21]. Despite this, studies have shown that among health care personnel there exists a negative attitude toward social media and a concern about patients seeking information online [22,23]. With the knowledge of what is actually discussed about introducing formula feeding on parenting Web forums, health care personnel can have a better understanding of the situation, and thus will be able to better adjust the breastfeeding support with the family’s needs.

The aim of this study was to analyze the perceptions of mothers, who are discussing the topic on Web forums, about introducing infant formula.

## Methods

### Design and Setting

This is a qualitative, descriptive, cross-sectional study conducted in 2015 using online data from 2 Swedish parenting Web forums. Much social interaction has been relocated to the Internet, and online data have thus become a good data source to capture experiences and views of people in the social and cultural contexts in which they appear [24,25]. The design was inspired by LiLEDDA, a 6-step forum-based ethnographic method for nursing research [26], which involves literature review and identification of research questions, locating the field online, ethical considerations, data gathering, analysis and abstractions, and trustworthiness.

### Sample

The participants in this study were the people posting their views on the 2 chosen online forums.

Most posters on these forums were anonymous, and therefore, we have little knowledge of the characteristics of the people who posted them. However, statistics provided by the websites show that most participants are women (88%), around 30 years of age, and who are either already a parent (61%), pregnant (14%), or planning pregnancy (5%). On the basis of how they expressed themselves in the posts, we were able to conclude that most were women with personal experience of breastfeeding.

### Data Collection

The forums were chosen because they were public forums that required no registration, they were easily accessible by large search engines, and they had a high frequency of postings, which make a meaningful analysis possible [26]. Following criteria were used for selecting the posts for the study: posts matching the aim, arguments for or against formula feeding and opinions about formula feeding, and opinions posted from August 2014 to August 2015. The posts could be shorter or longer and may consist of only 1 sentence or many sentences building a story. Posts concerning practical details about formula feeding were excluded. The data collection took place during September 2015, and the study was conducted by the second and third authors who searched through subject categories in the forums concerning infant feeding by using the search functions of the websites.

### Data Analysis

The data collection resulted in 107 pages of text that were analyzed by using Graneheim and Lundman’s [27] qualitative content analysis. Meaning units matching the aim were identified, condensed when necessary, and coded. A meaning unit could consist of a few words, 1 or several sentences, or even longer parts of the text, but should express the same content [27]. The meaning units were then interpreted and coded with shorter labels. These codes were then sorted and grouped into subthemes by comparing similarities and contrasts. This should not be seen as a linear process, rather a moving back and forth between the original text, meaning units, and codes. Finally, 3 themes that were identified formed a main theme, which described the latent content of the text. The analysis was made by the first, second and third authors, but to increase trustworthiness, all steps of the analysis and any uncertainties in coding and thematic sorting were discussed with the whole research team.

### Ethical Considerations

This study was exempt from ethical approval as the forums were public forums that did not require passwords or registration for access [26,28], and there were no policies on the websites forbidding the use of the information for studies. Informed consent from the posters was not obtained because the study was considered archival [26] and because posts were published publicly [24,28]. To protect the anonymity of the participants, the posts have not been identified and exemplifying quotes used are not traceable to the person who wrote those [26].

## Results

### Study Findings

Findings from the analysis is presented in Table 1 below as well as in text. In total, 370 posts resulting in 107 pages of text were analyzed. The analysis resulted in a main theme, “balancing between social expectations and confidence in your parental ability,” which has been derived from the following 3 themes: “striving to be a good mother,” “striving for your own well-being,” and “striving to discover your own path.” These 3 themes in turn have 3 subthemes each. The themes are presented in the text as headings, and subthemes are presented in italics directly in the text with exemplifying quotations.

**Table 1:** Main theme, themes, and subthemes.

Main theme	Themes	Subthemes
Balancing between the expectations of others and confidence in your parental ability	Striving to be a good mother	Breast is best
		Breastfeeding at any cost
		Bonding of importance
	Striving for your own well-being	Giving up due to pain and problems
		Feeling tied up—losing freedom
		Getting into control to feel secure
	Striving to discover your own path	Negotiating pros and cons
		Dealing with pressure and impact of others
		Standing up for your decision

### Striving to Be a Good Mother

In the forums, the assumptions that *breast is best* and that breastfeeding was the firsthand choice were often stated. Mother’s milk was frequently described as the best start for the child and being the most important during the first months. Breastfeeding recommendations were, according to some posts, considered as arguments not to introduce formula, whereas some participants questioned these recommendations and their validity for their child.

I try to stimulate my milk production, it feels like hard work but I trust that my milk is better for my children than formula.

The assumption that *breastfeeding should occur at any cost* and statements such as everyone who could breastfeed should breastfeed were expressed on the forums. Many had difficulty understanding why anyone would choose not to. Breastfeeding was sometimes described as a sacrifice that the mother should make for her child. On the other hand, some thought it could be acceptable not to breastfeed if the mother had strong enough reasons not to, but she should at least try before deciding not to.

I know that not everybody can breastfeed, but to choose not to without even trying that I cannot understand.

Women whose breastfeeding was not successful expressed that striving to breastfeed at any cost sometimes led to feelings of failure and inadequacy, and they felt that they were not good mothers. Giving formula could be associated with feelings of shame and guilt. Other women commented that one could be a good enough mother, even if the child received formula.

Talking about breastfeeding, it seems to be more important than anything else you do, I get the impression that breastfeeding is connected to being the “good mother” and in some way a measurement on how good a mother you are.

Online discussions about how bonding with the child would be affected by giving formula frequently took place. Although some discussions indicated that breastfeeding led to better bonding, others indicated that it did not influence *the bonding*. The natural aspects of breastfeeding and the resulting closeness to the child were stressed. However, other ideas were also expressed about whether the child would bond better with the father or partner when giving formula and that bonding with the mother would be negatively affected by breastfeeding when she was reluctant.

### Striving for Your Own Well-Being

The well-being of the mother was considered an important part of the decision to introduce formula. Many bloggers described problems with breastfeeding; sore nipples, mastitis, infections, and *giving up due to pain and problems* were common examples. Women struggled to give their child the best but many worried about how long they would have the energy.

I’m starting to wonder if it’s worth all the effort and bother I go through with it. It drains me of all my energy; I simply can’t even enjoy her like I want.

The participants often tried to normalize the breastfeeding problems of others by describing their own experiences and encouraging them to keep trying and not to give up. They also thought that previous difficult breastfeeding experiences would not necessarily mean that future experiences would be the same. In the forums, emotional and psychological aspects of breastfeeding and related stress were frequently discussed. Some felt uncomfortable when breastfeeding; women could also *feel tied up—losing freedom*. The opportunity for both parents to share the responsibility of feeding was sometimes seen as an advantage for giving formula. The woman could get relief and the partner could participate more in the care of the child.

I support bottle feeding if you find it mentally straining. It is easier to get assistance if bottle feeding is an option.

In the end, the main concern of many participants was that their child should be healthy and satisfied. Many posts pointed out that formula helped their children to sleep better, be more satisfied after the feeding, and gain weight better. Giving their child formula could be one way of *getting into control to feel secure*, for example, gaining control over their child’s intake.

Then I can see exactly how much [formula] my baby swallows and if it is always enough.

### Striving to Discover Your Own Path

In many discussions, *negotiations of pros and cons* were evident. Posts on the forums expressed concerns regarding the negative effect of formula on breastfeeding and concerns about how an early introduction of formula could interact with breastfeeding. Despite this, many comments encouraged partly breastfeeding when experiencing breastfeeding problems. Breastfeeding and formula were not always seen as conflicting. Some commented that children can grow and become healthy regardless of whether they had been raised on mother’s milk or on formula.

You never know how it will go or what you yourself will feel, or how things will work out for the baby. It will be okay no matter what, so take it easy and whatever will be, will be.

While making the decision to introduce formula, women had to *deal with the pressure and impact of others.* Health care personnel were sometimes seen as intrusive when they promote breastfeeding, and this could cause women to feel pressured and coerced into breastfeeding. Some described the personnel as being almost hysterical and sometimes almost violating the woman’s integrity.

I was exactly in the same situation as you! Everyone was getting at me, grabbing my breasts to get my breastfeeding going! Totally insane!

Some participants described that, after deciding to introduce formula, they no longer received support from the health care personnel. Concerns about the reactions from health care personnel were present both before and after birth. Pressure could also come from others in the woman’s surroundings. The experiences of others sometimes became arguments for whether the woman herself could breastfeed and influenced the choice of introducing formula. In the forums, contradictory advice was often given, which also created confusion and feelings of doing something wrong, no matter what you chose. Lack of understanding from family and friends could add to the feeling of guilt that the women had not lived up to expectations. However, examples of positive meetings with health care personnel could ease these feelings and justify their decision.

In the forums, women were often encouraged to *stand up for their decision* to introduce formula. Most posters considered feeding choices to be a personal decision of the mother. The woman should not have to justify herself or have a reason; it was enough to base it on her feelings and instincts. Some posts pointed out that the decision was up to the woman to make because it involved her body. However, it was still important that the decision should be based on facts and not arbitrary advice from others.

It doesn’t make any difference to me why a woman chooses not breastfeed. It is her own business. On the other hand, it would be interesting to know why so many women deliberately belittle each other’s choice with something as personal as breastfeeding.

## Discussion

### Principal Findings

The main theme in this study expresses how mothers are balancing between the expectations of others and their own confidence in their parental ability in relation to infant feeding. The 3 themes highlighted that the participants strived to be good mothers, but they also strived for their own well-being. To manage that, they strived to find their own path.

### Result Discussion

Breastfeeding was sometimes described as natural, whereas infant formula was characterized as a synthetic secondary alternative where women felt they had to justify the fact that the child was fed formula. There has been a link between infant feeding and the identity of a mother, and some felt that to be *a good mother*, one was supposed to breastfeed. This has been described in previous studies as failing to live up to womanhood and motherhood [29,30], perceptions of inadequate mothering [31], and feelings of having to defend the decision to feed formula to support their identity as a good mother [30,32].

The participants in our study expressed insecurity about doing right and being good enough mothers. They had to state valid reasons to give formula. Those who were unable or chose not to breastfeed had feelings of guilt and shame, something that has been confirmed by previous studies [29,31,33,34]. Taylor and Wallace [29] described how feelings of guilt were connected to giving formula for selfish reasons, such as putting your own needs before those of the child and thereby denying your child what is considered best. This notion of selfishness has been described as conflicting with the concept of being a good mother [32]. Giving formula for selfish reasons, such as not wanting to breastfeed, collides with the image of the self-sacrificing mother who gives endlessly of herself [33]. In addition to the feeling of guilt of doing something wrong when giving formula, women reported experiencing shame connected to failure, inadequacy, and not being a good mother [29,31]. Shame has been connected to social and cultural norms and “the right way” to feed your child [31].

In the Internet forums, many posts described how health care personnel reinforced the image of breastfeeding as a means of being a good mother, and thus inflicted guilt when women experienced breastfeeding problems and introduced formula. In breastfeeding promotion, focus often lies on the mechanism of transferring milk from mother to child and the nutritional benefits of human milk. Little is said about other aspects of the woman’s life that are affected. Women in our study sometimes expressed how they felt objectified in situations when health care personnel tried to support breastfeeding. If women’s desires and needs are belittled due to, for them, misguided focus, feelings of guilt and shame might arise. Today’s parenting culture is often expert-guided, primarily by the medical-scientific view that makes medical professionals authorities on defining risks and what constitutes a good choice [30]. According to Benoit et al [33], focusing on the biological aspects of breastfeeding exemplifies how health care personnel use their power to reinforce “the correct” maternal behavior. Primary focus on the biological aspects of breastfeeding diminishes the breastfeeding experience of the woman, making it physical instead of being a part of all aspects of her life. For instance, this can make women experience their breasts as a possession of their child instead of their own [33].

Women giving formula also need support and information as they have concerns about their child’s well-being. However, they also have their *own well-being* in focus to be *good mothers.* In our study, worries about the reactions from health care personnel were expressed and occurred when women had decided not to breastfeed. Sometimes, women even had difficulties bringing up the subject of formula feeding, something that has been described in previous studies [34,35].

Our results indicate that when health care personnel informed and supported women regarding formula feeding, their feelings of shame and guilt decreased. By opening the door to other options than merely breastfeeding, partial breastfeeding can also be promoted, which refers to our result about mothers’ need to *discover their own path*. Results from a previous study indicate that women who experience positive interactions with health care personnel breastfeed more than those who experience negative interactions [36]. Perhaps the conflict lies in the impossible equation of being the self-sacrificing, good mother and the informed, independent woman, which makes the issue of supporting breastfeeding more complex and stressful than just feeding a child. From literature, we know that stress is associated with the shorter duration of any and exclusive breastfeeding [37]. To promote breastfeeding without inflicting guilt and shame, health care personnel need to focus more on perceived stress and on the relationship between the mother and the child instead of the biological aspects of breastfeeding. They also need to be more open and listen to the woman’s desires and needs, take into account the amount of stress and her well-being in her particular situation, and support her in finding her own path, instead of assuming that all women should breastfeed.

### Study Limitations

This study was conducted in a Swedish context, where the social security system allows paid leave for parents for more than a year after birth. This environment is conducive to high breastfeeding rates and a maternity and child health care that can easily promote breastfeeding. The frequency of breastfeeding and norms surrounding breastfeeding might be typical of Sweden, and these circumstances can vary internationally and need to be taken in consideration when reading and reflecting about the transferability of our results.

The data collection was also conducted from online Web forums. On these forums, there are both active writers and passive readers. Our results mirror the perceptions of the active writers, and it is of course possible that the experiences of others would have been expressed if the silent readers had been studied, for example, in interviews. On the other hand, we view it as a strength that the participants could speak freely, without responding to a researcher’s questions. Therefore, we interpreted that the quotes were honest and based on inner perceptions and feelings.

### Conclusions

For women in early parenthood, it is important to feel that they are good mothers, to feel well, and also to find an individual path regarding breastfeeding. Feelings of guilt and shame may occur when women choose to formula-feed their children. To balance between the expectations of others and confidence in their own parental ability, women use Web forums for advice on whether to formula-feed or not. It could therefore be questioned whether health care personnel are fairly open about women’s different choices and often stressful situations. Therefore, Web forums are an important complement to health care. Parental Web forums could further be used by health care professionals to become familiar with women’s ambiguities.
